# Plant Functional Niches in Forests Across Four Climatic Zones: Exploring the Periodic Table of Niches Based on Plant Functional Traits

**DOI:** 10.3389/fpls.2020.00841

**Published:** 2020-06-17

**Authors:** Ruoyun Yu, Jihong Huang, Yue Xu, Yi Ding, Runguo Zang

**Affiliations:** ^1^Key Laboratory of Forest Ecology and Environment, State Forestry and Grassland Administration, Institute of Forest Ecology, Environment and Protection, Chinese Academy of Forestry, Beijing, China; ^2^Co-Innovation Center for Sustainable Forestry in Southern China, Nanjing Forestry University, Nanjing, China

**Keywords:** periodic table of niches, biomes, functional traits, convergence, niche, periodicity

## Abstract

Previous studies have indicated that a niche variation scheme, similar to the periodic table of elements, can be constructed based on functional traits. The periodic table of niches for species is defined as a multidimensional ordination scheme of niche relationships and their orders in a specific biotic community. Comparing functional trait-based niches is extremely useful in theoretical studies of plant ecological strategies, community assembly, and the geographic differentiation of biomes across different climatic zones. Here, data for 11 functional traits belonging to three fundamental niche dimensions (leaf economy, mechanical support, and reproductive phenology) were compiled for 215 woody species from forests across four climatic zones (tropical, subtropical, warm-temperate, and cold-temperate). We constructed the periodic table of niches based on the functional traits of plants in different communities and explored their variations among biomes. A principal component analysis (PCA) was performed to derive the dominant gradients of trait combinations for each individual niche dimensional dataset. Then species scores for the first two axes (PC1 and PC2) were used as inputs for a second PCA to ordinate species in the continuous niche space constrained by the three niche dimensions. Changes in the functional niches of plants from the four biomes along the PC1 and PC2 of niche space were examined based on species scores. Leaf economy was the dominant functional dimension in the plant niche space, followed by mechanical support. Considerable niche convergences among different species were found in the niche space for each biome, except cold-temperate forest. The species niches varied mainly with the increasing specific leaf area/decreasing stem tissue density along PC1, and with the decrease of leaf area/plant size along PC2 from tropical to temperate forests, suggesting that the ecological strategies of plants in the four biomes changed from conservative to acquisitive with an increase in latitude. Our results confirmed that the periodic table of niches does exist and can be constructed by major functional dimensions composed of dominant functional traits. The periodic table of niches effectively reflects the changes of ecological strategies of plant species in biomes across different climatic zones.

## Introduction

The fundamental goal of community ecology is to summarize the general rules of similar ecological phenomena (McGill et al., 2006). The niche is one of the core concepts in community ecology (Li et al., 2017; Datta et al., 2019). It plays a significant role in the study of mechanisms of species coexistence and the prediction of community assembly processes (Kirchheimer et al., 2018; Huang et al., 2019). The most influential definition of the niche may be the Hutchinsonian niche (Holt, 2009). Hutchinson proposed that the niche is a mapping of a space, with many axes that correspond to all possible environmental requirements of the species (e.g., temperature and nutrient supplies) (Hutchinson, 1957). Thus, numerous niche studies rely on environmental parameters related to species survival (Atwater et al., 2017; Procheş et al., 2019; Valdez et al., 2019). However, in natural communities, obtaining sufficient environmental niche axes for many species is hindered by practical constraints. The definition of the n-dimensional functional niche is analogous to the Hutchinson (1957) niche concept, except that the axes represent functional traits rather than environmental resources (Rosenfeld, 2002; Li et al., 2017). A trait-based analysis of niches may provide a better insight into community dynamics, because easily measurable functional traits not only co-vary with environmental gradients but also directly reflect the adaptive strategies of different species under varied living conditions (Violle and Jiang, 2009; Junker et al., 2019). Accordingly, ecologists have identified some patterns of niche variation based on functional traits that are strongly and consistently associated with environmental gradients (Smith et al., 2013). A novel niche study framework named ‘*the periodic table of niches*’ was developed to explore this problem (Winemiller et al., 2015).

**FIGURE 1:**
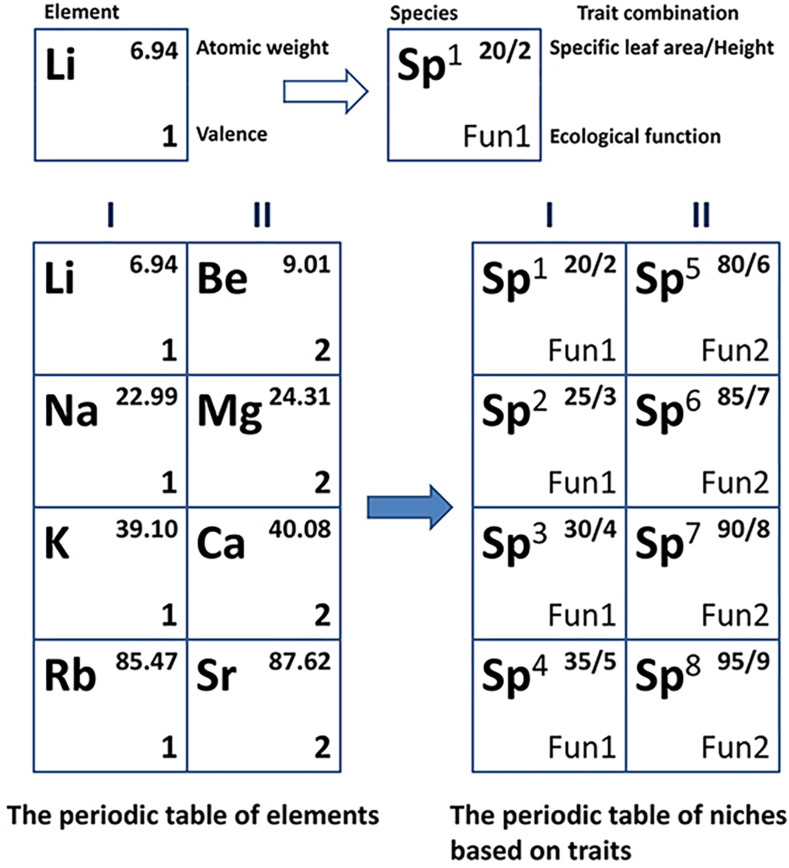
Conceptual analogy of the periodic table of niches to the periodic table of elements in a two-dimensional space. Column I of the periodic table of elements shows alkali metals and column II shows alkaline-earth metals. Analogically, species 1 to 4 in column I within the periodic table of niches are tropical evergreen shrubs, and species 5 to 8 are temperate deciduous trees.

The periodic table of niches for species is a periodic niche scheme that represents functional niche relationships and their orders in a specific biotic community (Winemiller et al., 2015; Pianka et al., 2017). The idea of such a niche table was first proposed by Pianka (1974) who was inspired by the periodic table of elements. The ecological features that support the construction of a niche scheme similar to the periodic table used in chemistry represent the convergent phenomenon of functional traits (Fukami et al., 2005) as well as trait co-variation across different environments (Reich, 2003; Kraft et al., 2008). The replication of functions across species from divergent families indicates the existence of periodicity (convergence) in niche space (Winemiller et al., 2015). In the same column of the periodic table, chemical elements with different electron shell numbers have the same number of outermost electrons and show similar chemical properties. Analogously, species in which some similar functional traits occur could also have essentially consistent ecological functions, just as a multitude of known convergences exist in many taxa across the globe. It should be noted that given the multidimensional nature of functional niches, the periodic table of niches is conceptually analogous to the periodic table of elements, but not structurally equivalent (see Figure 1 for details of the analogy).

The most primitive periodic table of niches is a two-dimensional scheme for terrestrial organisms based on trophic strategy and life history that was developed by Pianka (1974). Describing and comparing functional niches is not as simple due to their multidimensional complexity. A more comprehensive periodic table of niches was constructed to compare the diversification of lizard niches in relation to five basic dimensions: habitat, diet, life history, metabolism, and defense (Pianka et al., 2017). Species positions in relation to major functional gradients (body size, terrestrial versus arboreal microhabitat, and activity time) were explored within the continuous multidimensional niche space. Many convergent pairs of lizard species were also identified in the niche space. Researchers have used the periodic table of niches to facilitate comparative studies, such as investigations of niche convergence and spatial variation in community structure (Winemiller et al., 2015; Pianka et al., 2017). To date, this interesting and meaningful niche framework has only been used in animal systems. To the best of our knowledge, no study has constructed a periodic table of niches for natural plant communities. Such a periodic table of niches would facilitate the analysis of species relationships within the multivariate niche space (Pianka et al., 2017). The table would be unique and very different from the common multi-dimensional spatial assessment of plant traits, although the aim of both approaches is to predict community assembly and plant responses to ecosystem changes. Traditional trait research has mostly focused on functional trade-offs that underpin ecological strategies and the identification of species attributes that are responsible for those trade-offs (Reich, 2014). In addition to these benefits, the periodic table of niches approach tends to explore general rules, i.e., the periodicity in ecological phenomena, and to distinguish patterns of species niche diversification, while also summarizing the disparate ecological classifications developed for diverse taxa, habitats, and biomes. Therefore, extending the periodic table of niches to plant communities could lead to a better understanding of community assembly based on plant functional niches.

It should be noted that because of the huge differences in survival strategies between plants and animals, the dimensions of animal niches are not suitable for the exploration of plant functional niches. There is a need to determine which niche dimensions are appropriate for the trait-based niche space of plants. Considering that more than fifty functional traits of multiple plant organs are ecologically meaningful (Pérez-Harguindeguy et al., 2013), the dimensions of plant functions may be enormous. However, ecologists have proposed that many plant traits are redundant and we need to focus more on a limited number of niche dimensions (Laughlin, 2014). The most basic niche dimensions must represent the essential ecological strategies shaped by functional traits (Ackerly, 2004; Laughlin et al., 2010). Violle et al. (2007) defined ‘functional traits’ as ‘morpho-physio-phenological traits, which impact fitness indirectly via their effects on growth, reproduction, and survival.’ (Violle et al., 2007). Here, we summarize three key dimensions of functional niches based on plant functional traits: leaf economy, mechanical support, and reproductive phenology (Table 1), which have been frequently discussed in previous studies (Westoby, 1998; Diaz et al., 2016; Messier et al., 2017). Laughlin (2014) recommended studying traits from multiple organs (especially leaf, stem, and root) and considering flowering traits, given their consistent performance in explaining community assembly across different ecosystems (Laughlin, 2014). The well-known plant strategy scheme ‘Leaf-Height-Seed’ (LHS) is defined by specific leaf area, height, and seed mass, and quantifies pant ecological strategies in association with the leaf economics spectrum, competitive ability, and reproductive capacity (Westoby, 1998). However, functional dimensions are considered as gradients in suites of traits rather than gradients in only a single trait. The leaf economics spectrum is a useful framework for elucidating the trade-offs between fast/acquisitive to slow/conservative plant investment strategies, which synthesize multiple leaf traits that involve two key resources (carbon and nutrients) (Wright et al., 2004). Reich (2014) extended the idea of the leaf economics spectrum to stems, roots, and entire plants. The functional traits in stem tissues (e.g., stem tissue density) are recognized as powerful indicators of plant mechanical strength and construction costs (Messier et al., 2017). Plant size is also associated with mechanical support. Because larger plants generally fail in two ways, i.e., by uprooting or by trunk breakage, they are more vulnerable to wind action (Niklas, 2007). An analysis of the global spectrum of plant function revealed that plant size is a major dimension reflecting a plant’s ability to obtain resources (Diaz et al., 2016). In addition to the above traits of leaf economy and mechanical support, phenological traits, e.g., the time and duration of flowering, have a large role in the functional strategies of plant species. They are critical and can greatly influence plant reproductive success and fitness (Jensen et al., 2019). A multi-trait test suggested that flowering phenology fitted into the LHS scheme and was closely related to plant height (Laughlin et al., 2010). In the ponderosa pine forest, tall species flower late in the growing season.

**TABLE 1:** Functional traits and their assigned strategies within three functional niche dimensions.

**Niche dimension**	**Functional trait**	**Abbrev**	**Unit**	**Strategy**	**References**
Leaf economy	Leaf area	LA	cm^2^	The most common metric for leaf size, has important consequences for leaf energy and water balance	Diaz et al., 2016; Wright et al., 2017
	Leaf dry-matter content	LDMC	g g^–1^	A trait associated with the amount of structural material and stress tolerance of leaves	Zhao et al., 2016; Bruelheide et al., 2018
	Specific leaf area	SLA	cm^2^ g^–1^	The indicator of light capture and growth rate of plants	May et al., 2013; Reich, 2014
	Leaf nitrogen concentration	LNC	g kg^–1^	Nutritional quality of plants, closely related to the mass-based maximum photosynthetic rate and leaf life spans	Garnier et al., 2001; Wright et al., 2004
Mechanical support	Stem tissue density	STD	g cm^–3^	A key predictor of plant mechanical strength and construction costs	Chave et al., 2009; Messier et al., 2017
	Maximum plant height	MPH	m	The important parameters with respect to plant size and biomass, reflecting the self-supporting ability of species and their competitiveness for resources	Niklas, 2007; Rees et al., 2010; Iida et al., 2014
	Maximum diameter at breast height	MDBH	cm		
Reproductive phenology	Mean flowering time	FLT	day	Phenological traits that greatly influence plant reproductive success and fitness	Singh and Kushwaha, 2006; Rafferty and Nabity, 2017; Wolf et al., 2017
	Mean fruiting time	FRT	day		
	Flowering duration	FLD	day		
	Fruiting duration	FRD	day		

A periodic table of plant niches that integrates traits across leaf economy, mechanical support, and reproductive phenology could be used effectively to examine the variations and relationships of plant functional niches. In the niche-based process of community assembly, plant species with appropriate functional traits are screened into local communities by environmental filtering, leading to trait convergence (May et al., 2013). Climate is generally considered to be the most influential factor in environmental filtering (Frenette-Dussault and Hingrat, 2013; Shiono et al., 2015; Le Bagousse-Pinguet et al., 2017). Because environmental filtering is the pivotal driver of community assembly, species in diverse biomes under different climatic backgrounds must have specific niche characteristics. However, many studies of functional niches have focused on a single biome (Kraft et al., 2008; de la Riva et al., 2017; Jiang et al., 2018). There is relatively little information on the changes of functional niches across different plant biomes. In this study, we explored the periodic table of niches based on the functional traits of woody plants in forests across four biomes (tropical rainforest, subtropical evergreen-deciduous broadleaved mixed forest, warm-temperate coniferous-broadleaved mixed forest, and cold-temperate coniferous forest) in different climatic zones. These biomes vary significantly with the latitudinal gradient from south to north in China. We hypothesized that: (i) leaf economy is the core functional dimension of plant niche space and determines the primary ecological strategies of plant species across different biomes; (ii) the functional niches of plants across different biomes vary along dimensional gradients and are constrained by the dominant functional traits; and (iii) species ordination in the functional niche space indicates the existence of niche periodicity.

## Materials and Methods

### Study Sites

We selected four typical biomes in China to establish permanent forest dynamics plots (FDPs). The biomes were: (A) a tropical rainforest located in Bawangling Nature Reserve in Hainan; (B) a subtropical evergreen-deciduous broadleaved mixed forest located in Mulingzi and Xingdoushan Nature Reserve in Hubei; (C) a warm-temperate coniferous-broadleaved mixed forest located in Xiaolongshan Nature Reserve in Gansu; and (D) a cold-temperate coniferous forest located in Kanasi Nature Reserve in Xinjiang. The four study sites were distributed along a significant latitudinal gradient from 18°52′ N to 49°11′ N, and covered the longitudinal range from 86°54′ to 110°17′ E. The mean annual temperature decreased along the latitudinal gradient from 23.6 to −0.2°C. The mean annual precipitation ranged from 800 to 1,750 mm. Details of the study sites are shown in Table 2.

**TABLE 2:** The geographic profiles of the four typical biomes.

**Biomes**	**Location**			**Climate**	
	**Nature Reserve**	**Latitude**	**Longitude**	**MAT**	**MAP**
Tropical rainforest	Bawangling	18°52′–19°12′ N	108°53′–109°20′ E	23.6°C	1,750 mm
Subtropical evergreen-deciduous broadleaved mixed forest	Mulingzi and Xingdoushan	29°55′–30°10′ N	108°57′–110°17′ E	15.5°C	1,733 mm
Warm-temperate coniferous-broadleaved mixed forest	Xiaolongshan	33°30′–34°49′ N	104°22′–105°43′ E	10.9°C	800 mm
Cold-temperate coniferous forest	Kanasi	48°35′–49°11′ N	86°54′–87°54′ E	−0.2°C	1,065 mm

**MAT, mean annual temperature; MAP, mean annual precipitation.**

### Sampling Design

We randomly established fifty 20 m × 20 m FDPs in natural old-growth forests at each of the four study sites according to the standard protocols of the Center for Tropical Forest Science (Condit, 1998). In each FDP, we investigated all self-supporting woody plants with a diameter at breast height (DBH) ≥ 1 cm and recorded their height and DBH. Lianas were excluded, because their niches were strongly dependent on the self-supporting host trees (Muthuramkumar and Parthasarathy, 2001; Perez-Salicrup et al., 2001). Trees and shrubs were identified to species level with the help of local botanists. At least 10 robust individual plants per species were sampled to measure their functional traits. Only species with an abundance >20 individuals were sampled, covering over 90% of all individuals in each of the 50 FDPs. We sampled 215 species across the four biomes, belonging to 116 genera and 55 families. In eight cases, the same species occurred at two sites and their traits were, respectively, measured at each site. For each sampled individual, five fully expanded and healthy leaves and a 1–2 cm diameter branch were collected for trait measurements.

### Functional Traits

We measured seven functional traits for each species and collected four phenological traits from an online database^1^, the ‘Flora of China.’ We divided the 11 functional traits into three subsets based on their logical associations with three functional niche dimensions, which represented vital plant ecological strategies in terms of resource acquisition, mechanical support, and reproductive function (Table 1). The leaf area, leaf dry-matter content, specific leaf area, and leaf nitrogen concentration were the traits included in the dataset of leaf economy. The dataset of mechanical support consisted of stem tissue density, maximum plant height, and maximum diameter at breast height. Phenological traits were placed in the dataset of reproductive phenology, containing the mean flowering time, mean fruiting time, flowering duration, and fruiting duration.

Measurements of leaf traits followed the standard methods proposed by Pérez-Harguindeguy et al. (2013). We measured leaf area using the LI-COR3100C area meter (LI-COR, Lincoln, NE, United States). After weighing the leaf fresh mass, leaves were then dried for 72 h in an oven at 60°C to obtain the leaf dry mass. We calculated leaf dry-matter content as the ratio of leaf dry mass to fresh mass. The specific leaf area was then calculated as the ratio of leaf area to leaf dry mass. We measured the leaf nitrogen concentration using Kjeldahl digestion, followed by a colorimetric analysis. To characterize stem tissue density, one 5 cm long segment was cut from branches that had a diameter between 1 and 2 cm. We removed the pith, phloem, and bark, and then measured fresh volume using a Mettler–Toledo balance. The dry mass of the branch segment after drying for 72 h at 70°C was also measured. We calculated stem tissue density as the dry mass divided by fresh volume based on the entire sectional area (Pérez-Harguindeguy et al., 2013). Maximum plant height and diameter at breast height were calculated as the 95th percentile value for each species, based on at least 20 individuals across 50 FDPs in each study site (Fauset et al., 2015).

The phenological time and duration for each species were determined according to the Flora of China, which provided the first and last month in which a species is in flower or fruit. We used the Julian date to calculate the mean flowering time, mean fruiting time, flowering duration, and fruiting duration. For example, *Lithocarpus henryi* flowers from August to October, which corresponds to Julian days 213 to 304. Therefore, the mean flowering time = (213 + 304)/2 = 259, and the flowering duration = 304 − 213 = 91 days (Laughlin et al., 2010). The calculation of fruiting traits was the same as that of flowering traits. The methodology was constrained by the fact that phenological traits were not measured but obtained from a database, leading to some variance among species and between sites. Based on previous studies, the collected data were still considered reliable for quantifying phenological variations (Laughlin et al., 2010; Mo et al., 2017; Rafferty and Nabity, 2017).

### Data Analyses

**FIGURE 2:**
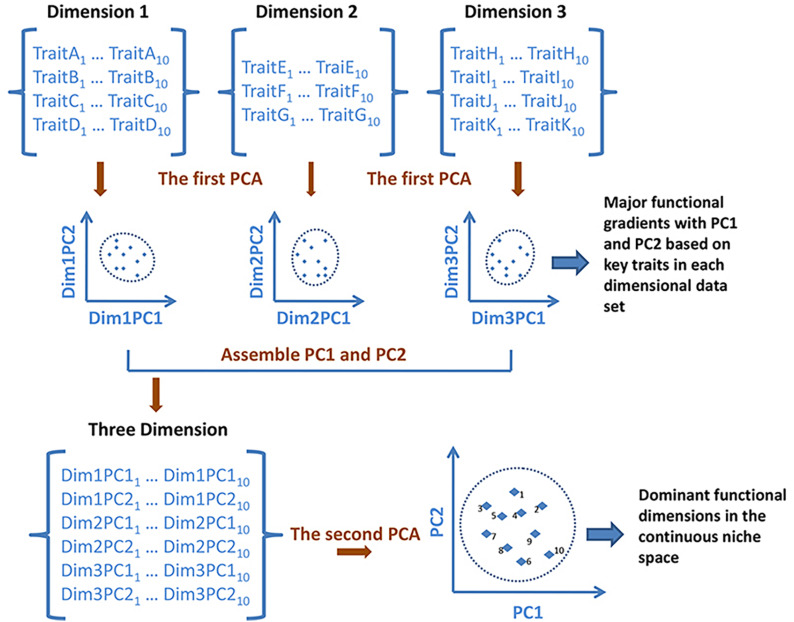
Schematic showing the creation of the continuous niche scheme by the ‘principal component analysis (PCA) of PCAs’ methodology. Dimension 1, dimension 2, and dimension 3 are trait data matrices associated with three different niche dimensions involving a set of ten species. Dim1/2/3PC1 and Dim1/2/3PC2 are the dominant gradients of trait combinations derived from the first PCA analyses performed on each individual dimensional dataset. The continuous scheme was derived from the second PCA using the species scores for the first two axes from the previous PCA of each dimension as input data.

Functional trait data were log10-transformed to meet the assumption of normality. Following the methodology of Winemiller et al. (2015), a principal component analysis (PCA) based on Euclidean distances was performed individually on each of the three dimensional datasets to achieve data reduction and produce the main gradients (i.e., principal components) of each dataset (Winemiller et al., 2015). Then, we input the species scores from the first two principal component axes for each dimensional dataset into a second PCA to create the niche ordination space, which is an ecological analog to the periodic table of elements (see Figure 2 for the calculation). This space, based on the ‘PCA of PCAs’ (Winemiller et al., 2015), was a continuous ordination of species relative positions within the multidimensional space that integrated the three functional niche dimensions, containing the 11 functional traits. Interpretation of the gradients in the final niche space was dependent upon the interpretations of gradients obtained previously from the first PCA of each individual dimensional dataset.

We performed the ‘PCA of PCAs’ to create the niche space, with a single biome. The key functional dimension was identified for each plant niche space. Periodicity in niche space was represented by distantly related species, which were tightly clustered around similar positions (Winemiller et al., 2015). To prove the existence of the periodicity/convergence of species niches, we calculated the ratio of the family number to species number in the same plot of each niche space. The eight selected plots (0.5 PC scores per plot, plots I–VIII) were all centered close to the origin in each niche space, because the plots around the edge of the space often contained only one species, with no obvious periodicity. A higher ratio meant that more distantly related species from different families converged into the same niche plot, and had a stronger convergence. Finally, we compared the niche differences of the four biomes in the same model. We tested whether the species functional niches represented by species scores of the first and second PC axes differed significantly between different biomes in the total niche space encompassing all 215 species, using a rank-based Kruskal–Wallis test. All analyses were carried out using R 3.6.0 (R Core Team, 2019) and the ‘vegan’ (Oksanen et al., 2019) and ‘agricolae’ (De Mendiburu, 2009) packages.

## Results

### Functional Niches, Functional Niche Dimensions, and Periodic Table of Niches in Each Biome

The main niche gradients represented by the key traits of each individual dimensional dataset (leaf economy, mechanical support, and reproductive phenology) were obtained by the first PCA (Table 3). These gradients retained most of the variation in the dataset. In the niche dimension of leaf economy, species from different biomes were all arranged along the gradient of specific leaf area, which was strongly correlated with PC1 (43.84–66.29%). The leaf area for tropical and subtropical forests, and the leaf dry-matter content for temperate forests were heavily loaded on PC2 (21.00–30.56%). In the mechanical support dimension, PC1 (94.78–98.95%) was associated with plant size, as represented by the maximum plant height and diameter at breast height, while PC2 was mostly related to stem tissue density and captured 2.20–3.36% of the variation. In the reproductive phenology dimension, PC1 (39.99–57.19%) was dominated by phenological duration (flowering and fruiting duration), while PC2 was related to the mean flowering and fruiting time, and accounted for 26.22–30.51% of the phenological variation.

**TABLE 3:** The percentage variation captured by the first two principal component (PC) axes from a principal component analysis (PCA) performed on each of the datasets with three niche dimensions.

**Niche dimension**	**First two PCs**	**TF PC 1**	**TF PC 2**	**SF PC 1**	**SF PC 2**	**WF PC 1**	**WF PC 2**	**CF PC 1**	**CF PC 2**
Leaf economy	Percent	43.84	27.57	53.52	23.74	55.32	21.00	66.29	30.56
	Major eigenvector	SLA	LA	SLA	LA	SLA	LDMC	SLA	LDMC
Mechanical support	Percent	96.51	2.20	94.78	3.10	95.34	3.36	98.95	0.91
	Major eigenvector	Plant size	STD	Plant size	STD	Plant size	STD	Plant size	Plant size
Reproductive phenology	Percent	39.99	28.27	46.28	30.51	42.50	28.12	57.19	26.22
	Major eigenvector	Duration	Time	Duration	Time	Duration	Time	Duration	Time

**TF, tropical rainforest; SF, subtropical evergreen-deciduous broadleaved mixed forest; WF, warm-temperate coniferous-broadleaved mixed forest; CF, cold-temperate coniferous forest. SLA, specific leaf area; LA, leaf area; LDMC, leaf dry-matter content; Plant size, maximum plant height and diameter at breast height; STD, stem tissue density; Duration, flowering and fruiting duration; Time, mean flowering and fruiting time. The major eigenvectors dominate the functional gradient of the PC axis*.*

**FIGURE 3:**
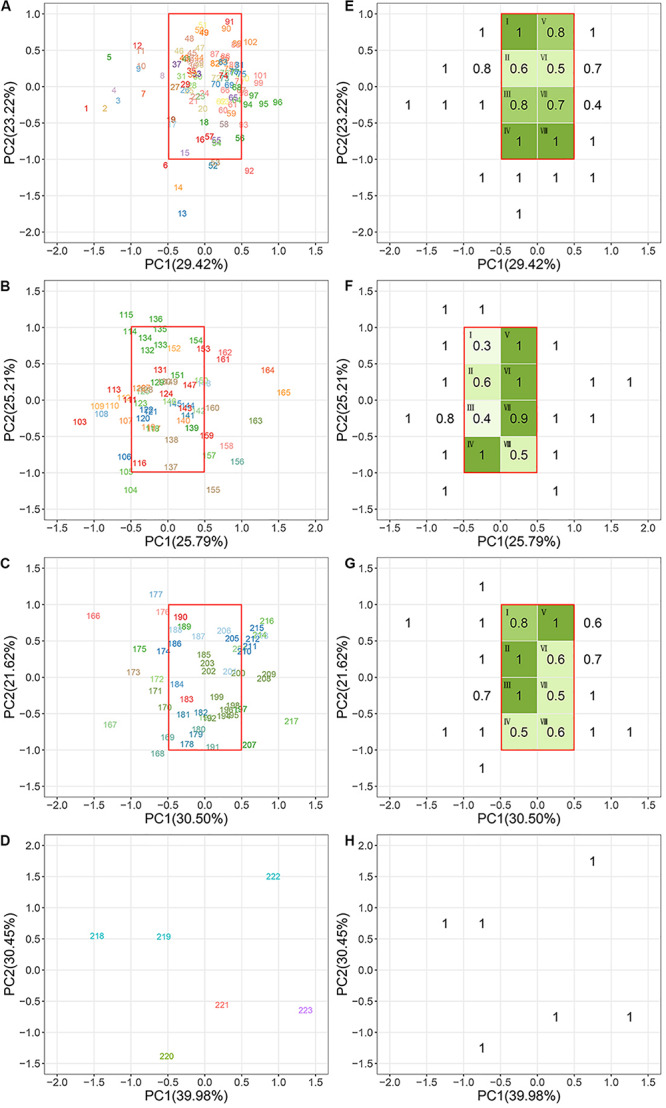
Species ordination within the continuous niche space in each biome. **(A,E)** Tropical rainforest; **(B,F)** subtropical evergreen-deciduous broadleaved mixed forest; **(C,G)** warm-temperate coniferous-broadleaved mixed forest; **(D,H)** cold-temperate coniferous forest. Panels **(A–D)** show the niche orders of plant species in each forest. Panels **(E–H)** show the niche convergences of plant species in each forest. The different colors of the species number indicate that species belong to different families. The species assembled in the same green plots (I–VIII) have convergent functional niches. Higher ratio values represent a stronger convergence. All species names are listed in Supplementary Table S1. Species names of plants in the same green plot of each biome are listed in Supplementary Table S2. See Table 4 for a more detailed description of the niche variation modeled by the first two PC axes of each niche space.

Species scores on the PC1 and PC2 from the second PCA were plotted to ordinate species within a continuous two-dimensional space of plant niches for each biome (Figure 3). In the niche space of the tropical forest, the PC1 was first dominated by pc_1_.leaf economy/specific leaf area (Table 4), which was also the main functional gradient along PC1 in the niche spaces of subtropical and cold-temperate forests. In the niche space of the warm-temperate forest, the pc_2_.leaf economy/leaf dry-matter content was more important than pc_1_.leaf economy/specific leaf area along PC1. The pc_2_.mechanical support/stem tissue density was heavily loaded on PC1 of the subtropical and warm-temperate forests. As with PC1, PC2 of the four niche spaces was also closely aligned to the leaf economy dimension. In addition, PC2 in the niche space of subtropical, warm-temperate, and cold-temperate forests all correlated with pc_1_.mechanical support/plant size. These results showed that leaf economy was the most critical functional dimension in the niche spaces of these four forests, followed by mechanical support. Considerable functional niche convergences were found in each niche space of the different biomes (Figures 3E–G), except the cold-temperate forest (Figure 3H). The niche convergences of distantly related species in the same niche occupations supported the existence of niche periodicity.

**TABLE 4:** Niche variation as modeled by the first two principal component (PC) axes of niche spaces for each biome based on the ‘principal component analysis (PCA) of PCAs’.

	**TF biome**		**SF biome**		**WF biome**		**CF biome**	
PCs	PC 1	PC 2	PC 1	PC 2	PC 1	PC 2	PC 1	PC 2
Eigenvalues	1.77	1.39	1.55	1.51	1.83	1.30	2.40	1.83
Percent	29.42	23.22	25.79	25.21	30.50	21.62	39.98	30.45
Eigenvectors								
pc1.leaf economy	**1.66**	−0.22	**1.32**	−0.48	−1.01	**0.95**	**0.82**	−0.30
pc2.leaf economy	0.39	**1.65**	0.80	**1.12**	−**1.07**	−0.85	0.27	**0.87**
pc1.mechanical support	0.95	−0.82	0.84	**1.18**	−0.98	−**0.87**	−0.02	**0.84**
pc2.mechanical support	−1.29	−**1.13**	−**1.27**	0.69	−**1.04**	0.87	−**0.77**	−0.23
pc1.reproductive phenology	−**1.33**	0.69	0.18	0.66	−0.46	−0.76	−0.51	0.27
pc2.reproductive phenology	0.05	−0.72	−0.48	1.04	−0.96	0.26	0.76	−0.02

**TF, tropical rainforest; SF, subtropical evergreen-deciduous broadleaved mixed forest; WF, warm-temperate coniferous-broadleaved mixed forest; CF, cold-temperate coniferous forest. The interpretation of Table 4 depends on Table 3. For example, specific leaf area (SLA) is the main gradient of PC1 in the leaf economy dimension of a tropical rainforest (Table 3). Accordingly, the eigenvector ‘pc1.leaf economy’ of a tropical rainforest represents SLA (Table 4). In the same way, the eigenvector ‘pc2.leaf economy’ of a tropical rainforest represents leaf area (LA). Bold values indicate the leading eigenvectors of each PC axis*.*

### Variations of Functional Niches Across the Four Biomes

**FIGURE 4:**
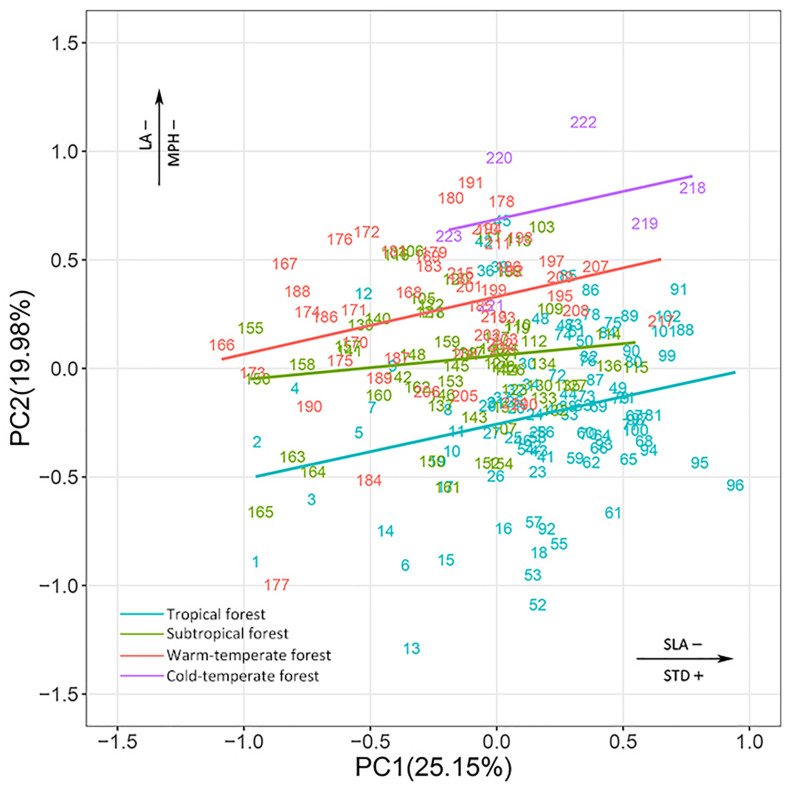
Species ordination within continuous niche space for species combined from the four biomes. The different colors of species number indicate that species belong to different biomes. All species names are listed in Supplementary Table S1. See Supplementary Tables S3, S4 for a more detailed description of the niche variation modeled by the first two PC axes of niche space.

**FIGURE 5:**
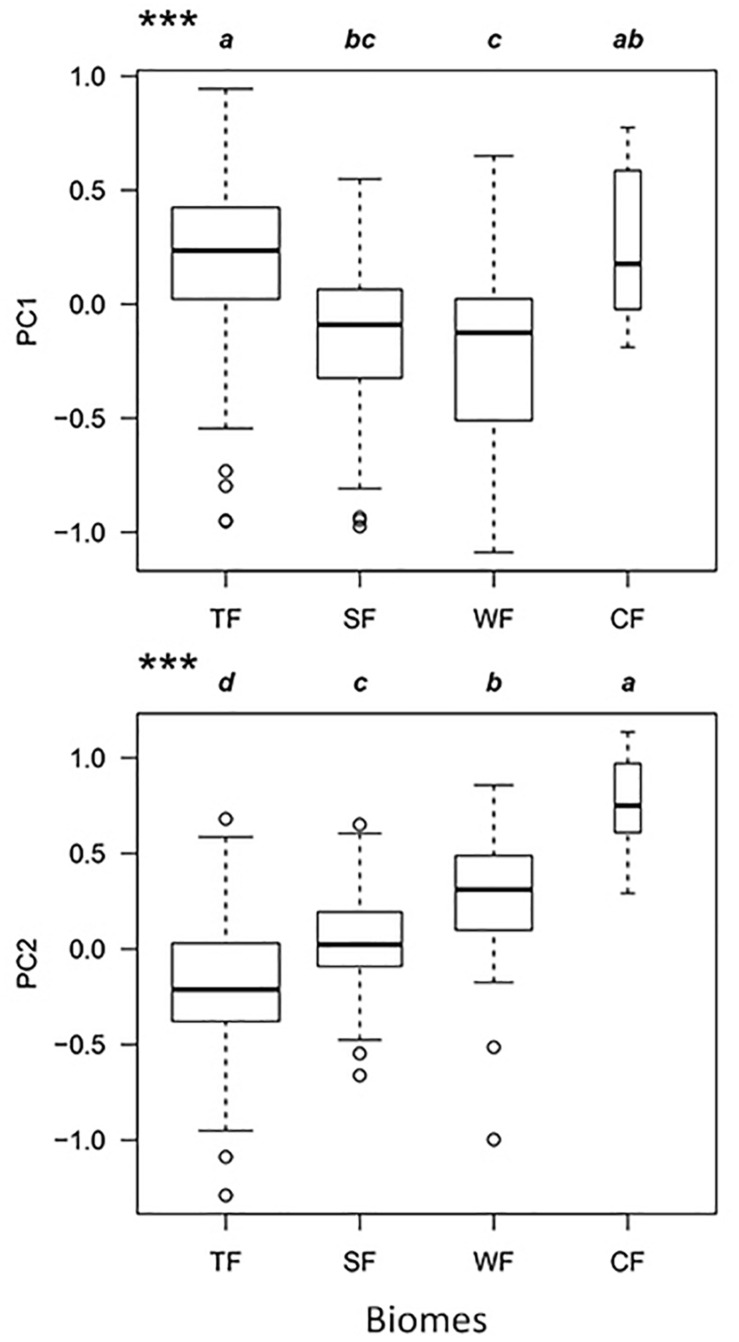
Functional niche variations of the combination of all species from the four biomes along PC1 and PC2 axes. TF, tropical rainforest; SF, subtropical evergreen-deciduous broadleaved mixed forest; WF, warm-temperate coniferous-broadleaved mixed forest; CF, cold-temperate coniferous forest. Significantly different contrasts (Kruskal–Wallis test) are indicated by different lowercase letters. ^∗∗∗^indicate significance at *P* < 0.001.

In the combined niche space of the 215 species from the four biomes, PC1 corresponded to pc_1_.leaf economy/specific leaf area and pc_2_.mechanical support/stem tissue density (Figure 4 and Supplementary Tables S3, S4). In contrast, PC2 represented pc_2_.leaf economy/leaf area and pc_1_.mechanical support/plant size. Species niches of different biomes had significant gradient differences from tropical to cold-temperate zones in the total niche space, especially along PC2 (Figure 5). Obvious changes in the plant functional niches occurred along these functional gradients (Figures 4, 5). As species scores on PC1 increased, species tended to have a lower specific leaf area and higher stem tissue density from cold-temperate to tropical forests. With increased species scores along PC2, the leaf and plant size of species became smaller from tropical to cold-temperate forests. An obvious spatial separation of tropical and temperate species was apparent in the niche space. Subtropical species, as transitional objects, respectively, converged to the tropical and temperate species within several plots in the upper left and lower right of the niche space.

## Discussion

### Dominant Functional Dimension and Variations in Plant Niche Spaces

Functional niches are complex and multidimensional. We studied plant niches from a trait-based perspective and constructed a niche scheme (i.e., an ecological analog of the periodic table of elements) that encompassed three fundamental dimensions: leaf economy, mechanical support, and reproductive phenology. The main functional gradients of the three niche dimensions across different biomes were identified according to their key traits. For example, specific leaf area dominated the principal gradient along the first PC axis of the leaf economy dimension, reflecting the variation in light capture and growth rate of plants (Zhao et al., 2016). The interpretation of the functional dimensions in the final niche space of each biome was dependent upon the gradients obtained previously from the PCAs of the three dimensional datasets. When integrating the results from each biome, we found that the most central functional dimension of plant niche space was leaf economy, especially when the specific leaf area was the dominant functional gradient. Specific leaf area was a significant indicator of a plant strategy based on different rates of resource acquisition and growth (‘fast and acquisitive’ or ‘slow and conservative’) (Reich, 2014). Species with a high specific leaf area commonly tend to have a high photosynthetic capacity and growth rate. Interestingly, unlike tropical, subtropical and cold-temperate forests, the leaf dry-matter content was more important than the specific leaf area in the niche space of warm-temperate forest. A possible explanation for this was derived. Of the four study sites, the comprehensive hydrothermal conditions were relatively poor in the warm-temperate forest (Table 2). Therefore, the leaf dry-matter content, which represents the stress tolerance of plants in harsh environments, was more sensitive to niche variations in the space of a warm-temperate forest (Pérez-Harguindeguy et al., 2013).

It should be noted that the stem tissue density and plant size associated with the dimension of mechanical support also had a great influence on the four niche spaces across the different biomes. Stem tissue density, which was heavily loaded on both the first and second PC axes, was more vital to plant size for the niche space of tropical forest. Stem tissue density is a powerful predictor of plant resistance to mechanical rupture (Messier et al., 2017). Tropical species are often threatened by typhoons and tropical forests contain many large trees. The variable stem tissue densities within these trees provide mechanical support, enabling plants to resist strong winds. However, the reverse is true for cold-temperate forests. Plant size represented the major change in niche space, being more significant than stem tissue density. The cold-temperate forest was located in an arctic climate region, with a long snow season and mean annual temperature of only −0.2°C. A dense stem is a disadvantage under a heavy snow load, which increases the possibility of mechanical breakage (Hacke et al., 2001). The six cold-temperate species in this study all had lower and stable stem tissue densities (the mean ± standard deviation of stem tissue density for cold-temperate and tropical species were 0.55 ± 0.03 and 0.60 ± 0.09 g cm^–3^, respectively), which minimized frost damage (Camarero and Gutiérrez, 2017). Plant size is related to the resource competition among species (Diaz et al., 2016), and it was found that four trees (e.g., *Larix gmelinii*) were more competitive than two shrubs (e.g., *Lonicera caerulea*) in the cold-temperate forest. Thus, plant size had a more important role than stem tissue density in the niche space of the cold-temperate forest.

Major differences in the plant functional niches were found in the niche space of the combination of all species from the four biomes. Plant species in the different biomes could be effectively determined according to their position along the main functional gradients (specific leaf area/stem tissue density for the first PC axis, leaf area/plant size for the second PC axis). As with specific leaf area, stem tissue density could be used as a marker of the position of a species along the fast-growing to slow-growing axis, which was related to the acquisition–conservation trade-off (Garnier et al., 2016). Previous studies have shown that plants with denser wood have lower growth rates (Messier et al., 2017). Tall trees also need a longer time to become established and their growth rate often declines as they become larger (Rees et al., 2010). Therefore, tropical species which are ‘lower specific leaf area, higher stem tissue density, larger plant size, (e.g., *Symplocos laurina* and *Beilschmiedia laevis*)’ have a conservative (slow) but safe strategy (de la Riva et al., 2017). Conversely, temperate species are ‘higher specific leaf area, lower stem tissue density, smaller plant size’, representing an acquisitive (fast) but risky strategy, (e.g., *Amelanchier sinica* and *Ostrya japonica*). Leaf area has considerable consequences for leaf energy and water balance (Diaz et al., 2016). The leaf sizes of tropical and subtropical species were larger than those of temperate species. The tropical and subtropical regions experience changeable hot and wet conditions. Accordingly, the leaf size of different species varied from <8 cm^2^ (*Ilex angulata*) to >800 cm^2^ (*Schefflera octophylla*) when adapting to such rich environments; however, the leaf size was smaller and less variable in temperate forests, and the leaves fall from the plants to reduce transpiration and retain water in the cold and dry seasons.

### The Periodicities of Plant Functional Niches

Traditionally in niche research, species location has been defined through the optimum environmental parameters required for species survival (Wright et al., 2006; Broennimann et al., 2012; Atwater et al., 2017). The periodic table of plant niches is an ecological analog of the periodic table of elements, which indicates species positions along the key functional axes of niches based on traits associated with plant ecological strategies, e.g., resource acquisition, support capacity, and reproductive fitness (Garnier et al., 2016). For example, based on the position of each species in the periodic table of niches, the general functional orders of plants can be identified, from huge leaves (*Pinanga discolor*) to needles (*Pinus koraiensis*), or from tall (*Cyclobalanopsis patelliformis*) to dwarf (*Lonicera caerulea*) plants. Changes of functional niches represent important trade-offs and adaptive strategies of plants in their growth and reproduction processes (Messier et al., 2017).

Niche periodicities have been verified in studies of fish and lizards (Winemiller et al., 2015; Pianka et al., 2017). The results of our study provide evidence that plant niches are also periodic. The strongest argument supporting the periodic characteristics of niches is convergent evolution (Winemiller et al., 2015). Classical community assembly theory suggests that habitat filtering is a widespread process driving the pattern of functional niches across plant communities (Li et al., 2017). This process results in an increase in the functional similarities of coexisting species, i.e., trait convergence. Considerable niche convergences were found in the niche spaces of tropical, subtropical, and warm-temperate forests. Pairs of distantly related plant species from different families were tightly clustered in similar niches. These repeated niche combinations across divergent plant taxa confirmed the existence of niche periodicity. For example, five species (*Prismatomeris tetrandra*, *Eurya loquaiana*, *Olea dioica*, *Symplocos ovatilobata*, and *Helicia cochinchinensis*) from different families converged into plot I of the tropical niche space. This indicates that these species have common functional responses to specific tropical environments. They all had a smaller leaf area (<35 cm^2^) and higher stem tissue density (>0.6 g cm^–3^). Comparatively, leaf area and stem tissue density were larger (>100 cm^2^) and lower (<0.5 g cm^–3^), respectively, for the species (e.g., *Polyalthia laui*, *Meliosma angustifolia*, and *Heynea trijuga*) in plot IV. It was not surprising that there was no periodicity of cold-temperate plant niches, as indicated by the spacious niche separation in the niche space of cold-temperate forest. In general, more gaps within the total realm of niche space would be expected in high-latitude communities than in those from lower latitudes that contained an abundance of species (Reinecke et al., 2016). This may be due to resource scarcity limiting the establishment of species that were not physiologically able to tolerate harsh abiotic constraints. As a consequence, niche separation is expanded when there are fewer species competing, allowing plants to persist in cold environments (May et al., 2013; Shiono et al., 2015).

Another argument for the periodicity of niches is that plant traits co-vary with the environment; hence, plant niches may exist along certain functional gradients and follow a general pattern under the process of environment filtering (Winemiller et al., 2015). The four study sites were distributed along a significant latitude gradient, with decreasing temperature and precipitation. The niche space of all plant species from the four biomes also featured significant functional gradient changes based on key trait combinations, which was a response to environmental changes. The species niches varied mainly with increasing specific leaf area/decreasing stem tissue density, and with the decrease of leaf area/plant size from tropical to temperate forests. The species niches of tropical and temperate forests were significantly separated in niche space. The individual species of each biome converged into their own niche space from the bottom to top along the functional gradients. Therefore, plants in each biome continued to emerge with specific functional niches (conservative or acquisitive), and then promoted community assembly in suitable environments. Thus, the periodic table of niches can be used to predict community progress in different biomes and to summarize the ecological principles of nature. The subtropical species had some tendency to converge to tropical and temperate species. The subtropical zone is a transitional climate zone. Similar functional niches were observed in different sites, further confirming the idea that a niche space exhibits periodicity, i.e., certain functional trait combinations are repeated across different plant biomes. It may be that in the transitional subtropical site, some sampling plots had a similar environment to the tropical or warm-temperate plots. Plants hence have convergent functional niches.

This study confirmed the utility of the periodic niche scheme proposed by Winemiller et al. (2015) and further developed it for use in plant communities. We analyzed plant traits for three key dimensions of functional niches; however, considering our limited trait sampling, these dimensions could not cover all aspects of plant ecological strategies. Our findings indicated that specific leaf area may be the most meaningful parameter for the study of trait-based community processes. Predicting community dynamics from specific leaf area is also very effective because it is easier and less expensive to measure than other functional traits (Garnier and Navas, 2012; Garnier et al., 2016). Given the advances in our results for woody plants and previous results reported for fishes and lizards (Winemiller et al., 2015; Pianka et al., 2017), we suggest that future research should extend this periodic table of niches scheme to other taxa (i.e., herbs or ferns), with the aim of providing insights into the orderly structures and functions of ecological science.

## Data Availability Statement

The datasets generated for this study are available on request to the corresponding author.

## Author Contributions

RZ designed the study. RY, JH, YX, YD, and RZ conducted the field investigation and collected the data. RY and JH performed statistical analyses and wrote the first draft. All authors contributed to the improvement of the manuscript.

## Conflict of Interest

The authors declare that the research was conducted in the absence of any commercial or financial relationships that could be construed as a potential conflict of interest.

## Footnotes

1 http://www.efloras.org
